# Spontaneous rupture of omental pseudoaneurysm in a patient on systemic anticoagulation

**DOI:** 10.1093/jscr/rjac511

**Published:** 2022-11-14

**Authors:** Gregory R Stettler, Jessica L Rauh, Meagan E Evangelista, Martin D Avery

**Affiliations:** Department of Surgery, Division of Trauma and Acute Care Surgery, Atrium Health Wake Forest Baptist Hospital, Winston-Salem, NC, USA; Department of Surgery, Atrium Health Wake Forest Baptist Hospital, Winston-Salem, NC, USA; Department of Surgery, Atrium Health Wake Forest Baptist Hospital, Winston-Salem, NC, USA; Department of Surgery, Division of Trauma and Acute Care Surgery, Atrium Health Wake Forest Baptist Hospital, Winston-Salem, NC, USA

## Abstract

A ruptured omental pseudoaneurysm is a rare cause of intra-abdominal hemorrhage. Herein, we present a case of bleeding ruptured omental pseudoaneurysm in a patient on systemic anticoagulation and successful treatment with surgery. A 72-year-old female on warfarin for atrial fibrillation presented with worsening abdominal pain. Cross-sectional imaging was obtained and was consistent with a large omental pseudoaneurysm (measuring 2.2 cm) as well as blood products within the abdomen. The patient was taken to the operating room where a pseudoaneurysm with evidence of active bleeding was identified. A diagnostic laparoscopy converted to exploratory laparotomy with partial omentectomy was performed. An omental pseudoaneurysm is a rare but potentially life-threatening cause of intra-abdominal hemorrhage. Given the risk of re-bleed, these lesions should be addressed promptly. In a facility that has the expertise, a catheter based approach with embolization may be considered, however, the mainstay of therapy should remain surgical resection.

## INTRODUCTION

Multiple etiologies of intra-abdominal hemorrhage exist and include traumatic injury, rupture of visceral aneurysms, neoplasia or complications of anticoagulant therapy. Omental pseudoaneurysms are one of the rarer etiologies and can lead to rupture with a potential for life-threatening intra-abdominal hemorrhage [1–5]. As omental pseudoaneurysms are rare, they are not frequently identified prior to intervention [3]. However, surgery has proven to be a durable treatment for this rare condition. Herein, we present a case of a bleeding ruptured omental pseudoaneurysm in a patient on systemic anticoagulation and successful treatment with surgery.

## CASE REPORT

**Figure 1:**
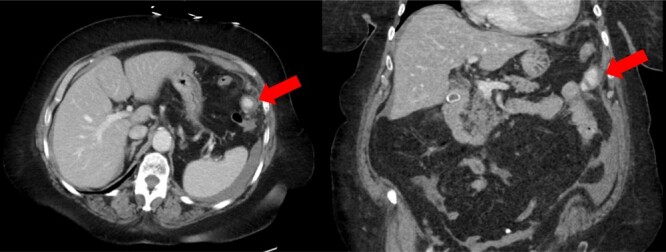
CT scan showing enhancing pseudoaneurysm (red arrow) with associated hemoperitoneum around the spleen, liver, and in left the paracolic gutter.

A 72-year-old female with a past medical history of chronic obstructive pulmonary disease, gastroesophageal reflux disease, hyperlipidemia, coronary artery disease and paroxysmal atrial fibrillation on warfarin (2.5 mg daily taken for several years) presented to a referring facility emergency department with worsening abdominal pain throughout the day. The patient had taken warfarin for several years without previous complications. Furthermore, she had no history of trauma or spontaneous bleeding. Her initial vital signs were unremarkable. Significant laboratory values included a hemoglobin of 13.0 and an international normalized ratio (INR) of 3.57, whereas all other labs were within normal limits. Cross-sectional imaging was obtained and the computed tomography (CT) scan revealed a left omental lesion measuring 2.2 cm with arterial and portal/venous enhancement concerning for a complex pseudoaneurysm (Fig. 1). Imaging also revealed a moderate volume (estimated 1000 cc) of hemoperitoneum. Given these findings, the patient was transferred to our facility for definitive care.

Upon arrival to our facility, the patient remained hemodynamically stable. Her warfarin was reversed with vitamin K as well as factor eight inhibitor bypassing activity (FEIBA) resulting in a normal INR level. The surgical service was consulted and due to need for long term anticoagulation, moderate volume hemoperitoneum and concern for rupture, the patient was taken to the operating room (OR) for resection of the presumed ruptured omental pseudoaneurysm.

In the OR, a laparoscopic approach was attempted given the patients stability. Upon insufflation of the abdomen, a moderate amount of hemoperitoneum was encountered. Further laparoscopic ports were placed to facilitate exploration. Although the site of the pseudoaneurysm could be seen, it could not be safely mobilized secondary to adhesive disease from previous abdominal wall hernia repairs. Furthermore, there was noted to be continued bleeding from the pseudoaneurysm. Conversion to an upper midline laparotomy facilitated direct visualization and finger pressure control of the bleeding pseudoaneurysm. The pseudoaneurysm within the omentum was mobilized and resected with an energy device and sent for pathologic evaluation. The abdomen was then irrigated and closed.

Post operatively, the patient recovered uneventfully and was discharged home on post-operative Day 3, after re-initiation of her oral anticoagulation therapy. Final pathology revealed a vessel with blood clot, histocytes and giant cells in the wall, which was consistent with a ruptured pseudoaneurysm. At her 2-week follow up in the clinic, she was continuing to recover well.

## DISCUSSION

An omental pseudoaneurysm is a rare cause of intra-abdominal pain and hemorrhage and infrequently identified prior to definitive intervention [1–4]. They are more common in men than in woman with a ratio of 6:1 [3]. Given reported cases of re-bleeding after initial conservative management, aggressive therapy for definitive treatment is preferred [3, 4]. Omental pseudoaneurysms are rarely idiopathic and often have an underlying predisposing factor. These include blunt or penetrating trauma, malignancy or complications of anticoagulant therapy, as is seen in this patient.

There are no specific recommendations for the treatment of pseudoaneurysms of the greater omental artery. However, recent recommendations from the Society of Vascular Surgery are to treat gastric, gastroepiploic, gastroduodenal and pancreaticoduodenal artery aneurysms or pseudoaneurysms of any size [6]. Furthermore, size criteria for repair of many visceral artery aneurysms is 2 cm or great [6]. Given a pseudoaneurysm is at greater risk of rupture than a true aneurysm [7], similar size criteria could be extrapolated to treatment of omental pseudoaneurysms. By location and size, this omental pseudoaneurysm should be treated.

When diagnosed on initial imaging, there are several therapeutic modalities that can be employed. Within the last several years, there have been multiple case reports within the Japanese literature describing transcatheter arterial embolization as treatment for omental pseudoaneurysms [2, 3]. The benefit of this approach being if is less invasive than surgery. However, the largest series of patients treated with an omental pseudoaneurysm comes over a 14 year period in Japan, of which 90% of patients underwent surgery, a majority of whom received an omentectomy. The remainder had either ligation or hematoma removal [3]. Similar to this Japanese series, our patient underwent an omentectomy. However, we employed a partial omentectomy given the left lateral location of the pseudoaneurysm within the omentum as well as the significant adhesions of the omentum from previous abdominal exploration. Furthermore, some authors recommend omentectomy over transcatheter arterial embolization, and even over ligation or hematoma evacuation, due to the possibility of malignancy associated with ruptured omental artery aneurysm or pseudoaneurysms [1]. In this patient, we believed that a surgical approach was warranted over a transcatheter approach given concern for ongoing bleeding and ability to expedite hemorrhage control by a surgical approach.

In about half of cases, patients may have their omental pseudoaneurysm initially misdiagnosed as an acute abdomen for another pathology, and only upon operative exploration are they correctly identified. In the other half of cases that are identified prior to intervention, less invasive techniques besides laparotomy can be considered [1, 3]. As in this case, a laparoscopic approach can be pursued. Only one other case of laparoscopic resection of omental bleeding has been reported [4]. However, as we experienced with this patient, if previous surgeries or ongoing bleeding preclude safe minimally invasive resection, a laparotomy should be promptly performed for control and resection of a bleeding pseudoaneurysm.

No specific recommendations exist for follow up or surveillance for resected greater omental pseudoaneurysms. For most visceral aneurysms, recommendations include one-time cross-sectional imaging to identify the presence of concomitant aneurysms. For embolized gastroepiploic lesions, recommendations for surveillance include imaging every 1–2 years, however, no recommendations exist following resection of these lesions [6]. Similarly, no data exist regarding recurrence rates of treated greater omental pseudoaneurysms. However, recanalization rates of endovascularly treated visceral aneurysms may be as high as 15% [6].

## CONCLUSION

An omental pseudoaneurysm is a rare but potentially life-threatening cause of intra-abdominal hemorrhage. Given the risk of re-bleed and rupture, these lesions should be addressed promptly. In a facility that has the expertise, a catheter based approach with embolization may be considered, however, the mainstay of therapy should remain surgical resection, especially in the setting of any sort of hemodynamic instability, concern for ongoing hemorrhage, or risk of underlying malignancy.
